# The essential role of peer relationships in students' motivation during adolescence

**DOI:** 10.1111/bjep.12772

**Published:** 2025-04-03

**Authors:** Fabian Schimmelpfennig

**Affiliations:** ^1^ University of Greifswald Greifswald Germany

**Keywords:** adolescence, motivation, peer relationships, self‐determination theory

## Abstract

**Background:**

Mid‐adolescence is a critical developmental stage during which peer relationships become increasingly important, while academic motivation tends to reach its nadir. Although positive peer relationships are known to promote students' motivation and that high motivation can benefit more positive social behaviours, no studies have examined this association reciprocally over time.

**Aims:**

Accordingly, this study aimed to test the potential reciprocal relationship between mid‐adolescent students' motivation and their perceived peer relationships in class by considering (a) different facets of peer relationships, (b) the peculiarities of peer relationships in high‐track schools and (c) the quality of motivation in a differentiated way.

**Sample:**

Questionnaire data from 779 high‐track students from Germany (*Range*_*age*_ = 12–15; 57% female) were used to test the interplay between students' perceptions of peers as positive and negative motivators, the student–student relationship and the quality of motivation (i.e., extrinsic, introjected, identified regulation, intrinsic).

**Method:**

A latent cross‐lagged panel model (CLPM) considering students' grades was run to examine the interplay.

**Results:**

The results of the CLPM show that students' perceptions of peers as positive motivators, as well as positive student–student relationships at the beginning of eighth grade, positively predict students' identified regulation at the end of ninth grade. In contrast, students' perceptions of peers as negative motivators negatively predict their identified regulation over time.

**Conclusions:**

Fostering peers as positive motivators in school might be beneficial for fostering quality motivation in students, particularly for identified regulation. Poor grades can encourage the tendency to let peers exert influence as negative motivators.

## INTRODUCTION

When individuals enter adolescence, their peers become more important both in the classroom (as student–student relationships) and outside of school (as general peer relationships) (Kilday & Ryan, 2022; Rodkin & Ryan, 2012). They can act as role models and socialization agents who meet the social needs of the growing child (Harter, 1996; Rubin et al., 2006, 2009). While student–student relationships refer to interactions in a structured educational setting, general peer relationships include friendships and broader social networks beyond school. Both types of peer interactions can either facilitate or hinder adolescents' motivation for schooling (Daumiller & Hemi, 2025; Kilday & Ryan, 2022; Kindermann, 2016; Rodkin & Ryan, 2012; Ryan & Shin, 2018; Wentzel, 2017; Wentzel et al., 2018). This is particularly essential in adolescence, as several studies have described a motivational decline in this developmental phase, with a low in ninth grade (Eccles & Wigfield, 2002; Fredricks & Eccles, 2002; Gnambs & Hanfstingl, 2016; Martin & Steinbeck, 2017; Tuminen et al., 2020; Watt, 2004).

Although it is known that positive peer interactions can promote student motivation (Kindermann, 2016) and that high motivation can benefit more positive social behaviours (e.g., Eccles & Wigfield, 2002; Fredricks et al., 2004; Hattie et al., 2020), few studies to date have looked at the interplay between (a) different facets of peer relationships, (b) the peculiarities of peer relationships in specific types of schools and (c) the quality of motivation in a differentiated way. To address this gap, the present study differentiates between student–student relationships and general peer relationships and examines their separate contributions to motivational development. Using a cross‐lagged panel model, we investigate the interplay between different types of peer interactions (peers as positive motivators, peers as negative motivators and student–student relationships) and different forms of motivation in the course of middle adolescence, from the beginning of eighth grade to the end of ninth grade, in students attending high‐tracking schools.

### Peer relationships in school

The term “peers” is often defined in the literature as a group of individuals who are at a similar age or stage of development and who interact in social contexts such as school, leisure or family (Rubin et al., 2006). “Peer relationships” therefore encompass social interactions and relationships between these peers, both within and beyond structured educational settings. These relationships are characterized by features such as symmetry and equality, which distinguish them from relationships with adults (Bukowski et al., 2018). Within the school setting, the term “students” specifically refers to individuals engaged in formal education, highlighting their role in structured learning environments (Wentzel & Muenks, 2016). While all students have peers, not all peers are necessarily students, as peer relationships extend beyond the classroom to include friendships and social networks outside of educational settings (Brown & Larson, 2009). This distinction is important for understanding how adolescents navigate social interactions both academically and personally, as peer influences may differ depending on whether they occur within structured school relationships or more informal social contexts (Ryan, 2011).

Social interactions with peers play vital roles in the lives of adolescents, acting as socializing agents and essential sources of social support (Brown & Larson, 2009; Veenstra & Laninga‐Wijnen, 2022). Positive peer interactions therefore support, as mentioned by SDT, basic psychological needs for relatedness, competence and autonomy (Ryan & Deci, 2000). As students navigate through this stage, adolescents are more inclined to seek emotional support beyond their family (Arnett, 2006; Boisvert & Poulin, 2016; Dishion & Tipsord, 2011; Portt et al., 2020; Rodkin & Ryan, 2012) and to develop closer and more intimate connections with their peers (De Goede et al., 2009; Rubin et al., 2006). Peers can act as role models, and individuals needing to “fit in” becomes particularly important during adolescence (LaFontana & Cillessen, 2010). Additionally, students form friendships based on similarities at the academic level, underscoring the appeal of academic similarity in forming social connections (DeLay et al., 2016; Gremmen et al., 2017). Adolescents often prefer study partners who are approachable, cooperative and friendly, and who are people they can learn from (Palacios & Berger, 2022; Palacios et al., 2024). For low‐achieving students, peers with similar performance levels are particularly attractive as friends, likely due to shared academic experiences and challenges. Conversely, high‐achieving students tend to avoid friendships with lower‐achieving peers, instead favouring connections with classmates who share similar academic interests and motivations, which helps to enhance their own academic success. Additionally, high achievers value friendships with individuals they enjoy spending time with and whom they trust to discuss personal issues. The influence of friendships on academic achievement becomes more evident as students grow closer and their connectedness fosters greater similarity in grades over time (Gremmen et al., 2017; Palacios & Berger, 2022).

### Student–student relationships in the classroom

The term *student–student relationships* refers specifically to interactions and relationships between students within the school context. These relationships are shaped by institutional roles and structures (e.g., group work, teacher instructions; Furrer et al., 2014). While peer relationships are often voluntary and informal, student–student relationships can also be influenced by classroom dynamics, academic competition or external pressures from teachers and school administration (Mercer & Howe, 2012; Wentzel & Muenks, 2016). However, while these external conditions may influence the formation of student–student relationships, their motivational effects depend on how they are subjectively experienced by students. If students perceive these relationships as supportive and autonomy‐enhancing, they are more likely to foster internalized motivation. Conversely, if such relationships are experienced as controlling or competitive, they may lead to external regulation.

In contrast to general peer relationships, student–student relationships describe how students interact within the classroom setting and how these relationships contribute to the learning process. Positive student–student relationships can foster cooperation, respect and mutual support (Bukowski et al., 2018; Wentzel, 2016), while negative student–student relationships can contribute to conflicts, exclusion or disengagement (Gest et al., 2014).

Studies have shown that positive student–student relationships support academic motivation (Baker et al., 2008; Hamre & Pianta, 2001; O'Connor et al., 2011), while negative experiences, such as peer rejection, have been linked to heightened absenteeism and reduced emotional engagement at school (Danneel et al., 2019; DeRosier et al., 1994; Kupersmidt & Coie, 1990). Notably, even transient or isolated instances of rejection (Greenman et al., 2009) have been linked to adverse academic consequences, and negative interactions with peers can lead to sustained disengagement (Buhs et al., 2006). This is particularly important in adolescence, when the importance of peer interactions inside and outside of school increases while, at the same time, school motivation tends to decline.

### Peers as motivators and their influence on motivation

In this study, peer relationships were defined using the Relationship and Motivation (REMO) scales (*Peers as positive motivators* and *Peers as negative motivators*), which capture the influence of friend groups on school motivation (Raufelder, Drury, et al., 2013). These scales extend the focus to include friendships both inside and outside the classroom. Friendships within the class can play a special role, as friends are embedded in the same social context, and these relationships are often associated with more intensive exchange and greater social closeness (Berndt, 2002; Hamm & Faircloth, 2005).

Positive peer relationships encompass a range of social interactions and qualities characterized by mutual acceptance, support, respect and prosocial behaviour (Rubin et al., 2006; Wentzel, 2016; Wentzel et al., 2016). These relationships can include both friendships and less‐close but constructive interactions (Bukowski et al., 2018; Newcomb & Bagwell, 1995). They promote emotional well‐being and social learning and contribute to the development of skills such as empathy, conflict resolution and teamwork (Bukowski et al., 2018; Wentzel, 2016).

In particular, positive peer relationships in school contexts can be defined as those that support cooperation, mutual learning and the integration of all students (Newcomb & Bagwell, 1995) as well as provide motivational support (Raufelder, Drury, et al., 2013; Raufelder, Jagenow, et al., 2013).

The distinction between *peer relationships* and *student–student relationships* is critical to understanding how different social dynamics contribute to motivation. While student–student relationships reflect classroom‐based interactions, *peers as motivators* capture an evaluative perception of peer influence on motivation. This study examines how both aspects interact with different forms of motivation and how their influence changes over time. This is particularly important in adolescence, when the importance of peers inside and outside of school increases for individuals while, at the same time, school motivation decreases for most students.

### Motivation

A longitudinal study of 3500 secondary school students in Germany has shown that motivation is more decisive than intelligence for academic success, especially in middle adolescence (Murayama et al., 2013). Greater motivation results in higher achievement and, consequently, fewer school dropouts (Kushman et al., 2000). The framework of self‐determination theory (SDT; Deci & Ryan, 1985) distinguishes the following forms of motivation: amotivation, extrinsic motivation (external regulation, introjected regulation, identified regulation, integrated regulation) and intrinsic motivation (Ryan & Deci, 2000).

Intrinsic motivation embodies autonomous behaviour driven by inherent satisfaction and epitomizes self‐determination (Ryan & Deci, 2000). For example, a student might engage in math homework out of pure enjoyment and curiosity about solving problems.

Extrinsically motivated behaviours span the spectrum between amotivation and intrinsic motivation, with varying degrees of regulatory autonomy. Externally regulated behaviours, the least autonomous, are driven by external demands or rewards and are often perceived as controlled or alienated (DeCharms, 1968; Skinner, 1953). For instance, a student might study for an exam solely to avoid punishment from their parents or to receive a promised reward. Introjected regulation involves adopting regulation without full internalization and is driven by factors like guilt avoidance or ego enhancement (Deci & Ryan, 1995; Nicholls, 1984). An example is a student who strives for good grades to avoid feeling guilty about disappointing their teacher.

More‐autonomous forms include regulation through identification, in which the behavioural goal is consciously valued, and integrated regulation, in which identified regulations align with one's values and needs (Deci & Ryan, 1995). For instance, a student might study diligently because they value education as a means towards achieving their future career goals (identified regulation) or because doing well academically aligns with their broader sense of identity and self‐worth (integrated regulation). Although they resemble intrinsic motivation, these forms remain extrinsic as they aim for separable outcomes rather than inherent enjoyment (Ryan & Deci, 2000).

Previous studies have shown that as students enter adolescence, their motivation tends to decrease and reaches a low point in ninth grade (Eccles & Wigfield, 2002; Fredricks & Eccles, 2002; Gnambs & Hanfstingl, 2016; Martin & Steinbeck, 2017; Tuminen et al., 2020; Watt, 2004). Not only does motivation itself decrease but also the quality of motivation (i.e., children are generally less intrinsically motivated and more externally regulated; Chen, 2013). Scientists have been searching for years for ways to break or mitigate this downward spiral. Since external impulses can facilitate or impede the internalization and integration of regulatory processes (Deci & Ryan, 1985), it has been shown that relationships with peers are an essential influencing factor in student motivation. While previous studies have shown that peers can influence motivation at school (Raufelder, Drury, et al., 2013; Raufelder, Jagenow, et al., 2013), only a few studies have differentiated motivation according to SDT (Beiswenger & Grolnick, 2009). Conversely, since highly motivated students also show more positive social behaviour (Kilday & Ryan, 2022), it is also conceivable that high motivation favours positive peer relationships.

### Motivation and peer relationships at high‐tracking schools

Building on Bronfenbrenner's ecological system theory, which emphasizes that the individual and their social environment mutually influence one another over time, we investigated how students' motivation and their perceived peer relationships (i.e., student–student relationships, peers as positive motivators and peers as negative motivators) influence each other over time. This perspective is particularly relevant for high‐tracking schools, where unique social and learning environments may shape students' motivational trajectories and academic outcomes such as grades.

According to Bronfenbrenner (1979), the microsystem, such as the classroom, represents a crucial context within which peer interactions directly influence student development. This dynamic aligns with Eccles and Midgley's (1990) stage–environment fit theory, which posits that the degree of alignment between adolescents' developmental needs and their environments significantly impacts their motivation, engagement and academic performance. In particular, grades at high‐tracking schools are not only an indicator of individual achievement but also play a key role in shaping social recognition among peers, which may further influence motivation and peer dynamics over time.

The German school system, with its early differentiation and the specific conditions at grammar schools, offers a unique opportunity to study the dynamics between peer relationships, motivation and academic performance. Grammar schools are characterized by a high level of achievement, strong competition and a close link between social recognition and academic performance (grades), making this type of school a relevant field of study (Baumert & Köller, 1998). At the same time, while peer relationships in this context significantly influence the development of learning and achievement processes, there has been little research on these interactions to date (Ryan, 2001).

Specifically, positive peer interactions within high‐tracking schools can foster higher externally regulated forms of motivation by supporting basic psychological needs for relatedness, competence and autonomy, as suggested by SDT (Ryan & Deci, 2000). Conversely, negative peer influences, such as pressure to disengage or to prioritize non‐academic pursuits, can undermine these needs, potentially leading to external or introjected regulation.

Additionally, identity development and peer influence mechanisms provide lenses through which to understand how motivation may be shaped by peer relationships. Brechwald and Prinstein's (2011) identity theory underscores the importance of social feedback from peers in shaping adolescents' self‐concepts and behaviours, suggesting that peer interactions can reinforce motivation through the alignment of group norms and personal goals. Bandura's social learning theory (Bandura & Walters, 1977) further emphasizes that students may adopt the motivational patterns they observe in their peers, particularly those whom they admire or perceive as role models. Finally, peer pressure (Veenstra & Laninga‐Wijnen, 2022) can function as a double‐edged sword, either encouraging engagement with academic tasks or promoting disengagement, depending on the group's values.

In the German context, high‐tracking schools represent distinct educational environments that amplify these dynamics. Prior research has shown that students at high‐tracking schools achieve higher academic performance (Baumert et al., 1996) and experience unique developmental opportunities (Neumann et al., 2007). However, the roles of peer relationships within these settings, particularly with regard to their influence on motivation over time, remain underexplored.

### The present study

While positive peer relationships can enhance student motivation (Kindermann, 2016) and high motivation can foster positive social behaviours (e.g., Eccles & Wigfield, 2002; Fredricks et al., 2004; Hattie et al., 2020; Kilday & Ryan, 2022), few studies have explored their dynamic interplay, specifically at high‐tracking schools. This study addresses this gap and examines how different forms of peer relationships (i.e., peers as positive motivators, peers as negative motivators and student–student relationships) interact with various types of motivation from adolescents in high‐tracking schools. The following hypotheses were tested.

Positive student–student relationships will be positively associated with higher forms of external regulation (introjected and identified regulation) over time, and these forms of motivation will, in turn, positively predict improvements in student–student relationships. However, no significant relationship is expected between student–student relationships and intrinsic motivation. *Rationale*: Supportive relationships within the classroom foster feelings of relatedness and competence, which are critical for the development of introjected and identified regulation (Ryan & Deci, 2000). However, intrinsic motivation arises entirely from within the individual and does not rely on external stimuli, even positive ones, making a connection to peer relationships unlikely (Ryan & Deci, 2000).

Peers as positive motivators will be positively associated with higher forms of external regulation (introjected and identified regulation) over time, and these forms of motivation will, in turn, positively predict greater perceptions of peers as positive motivators. However, no significant relationship is expected between peers as positive motivators and intrinsic motivation. *Rationale*: Motivational influence from peers reinforces the internalization of academic goals (Ryan, 2001). However, intrinsic motivation, as a completely self‐determined form of motivation, does not depend on external influences, even from supportive peers, aligning with the principles of SDT (Ryan & Deci, 2000).

Neither positive student–student relationships nor peers as positive motivators will be significantly associated with external regulation over time. Positive student–student relationships and positive motivators are expected to promote internalized forms of motivation (introjected and identified regulation) rather than compliance driven by external pressures. Although student–student relationships can be influenced by external structural factors (e.g., classroom hierarchies or school‐imposed group work), these factors primarily shape their formation rather than their motivational function. The extent to which student–student relationships promote internalized motivation depends on their qualitative nature and whether they are perceived as supportive and autonomy‐enhancing. *Rationale*: Positive motivators, such as supportive and encouraging peers, do not exert the kinds of external pressure typically associated with external regulation. Instead, they help foster internalized motivational states by aligning with personal values and goals (Ryan & Deci, 2000).

Peers as negative motivators will be negatively associated with higher forms of external regulation (introjected and identified regulation) over time, and these forms of motivation will, in turn, predict reduced perceptions of peers as negative motivators. No significant relationship is expected between peers as negative motivators and intrinsic motivation. *Rationale*: Negative peer influences undermine the fulfilment of psychological needs, leading to lower levels of introjected and identified regulation (Ryan & Deci, 2000). Intrinsic motivation, being self‐sustaining and independent of external factors, is not expected to correlate with negative peer influences (Ryan & Deci, 2000).

Peers as negative motivators will be positively associated with external regulation over time, and external regulation will, in turn, predict increased perceptions of peers as negative motivators. *Rationale*: Negative peer influence often leads to compliance driven by external pressures, such as pressure to avoid rejection or seek acceptance, aligning with the mechanisms of external regulation (Veenstra & Laninga‐Wijnen, 2022).

Higher academic grades will be positively associated with positive student–student relationships, peers as positive motivators and higher forms of motivation (intrinsic motivation, introjected and identified regulation). *Rationale*: High academic performance may enhance social status and approval among peers, reinforcing supportive relationships and internalized motivation (Ryan, 2001). Although intrinsic motivation is driven by internal satisfaction and personal interest, most intrinsically motivated students perform well in subjects that interest them (Ryan & Deci, 2000).

Lower academic grades will be positively associated with peers as negative motivators and external regulation. *Rationale*: Lower academic performance may lead to feelings of alienation and to engagement with peers who devalue academic success, thereby fostering external regulation and disengagement (Eccles & Midgley, 1990).

## METHOD

### Participants and procedure

The data was assembled in the fall semester at the start of the German academic year in 2011 (Time 1–T1) and in the spring semester at the end of the German school year in 2013 (Time 2–T2) in the frame of a longitudinal study (SELF) funded for 8 years by the Volkswagen Foundation. The total number of participants contained 779 eighth grade students at (*Rangeage* = 12–15, 57% girls) from thirteen high‐tracking schools randomly selected from a pool of 124 secondary schools in Brandenburg, Germany. Eight were located in rural areas, while five were based in Brandenburg's largest urban areas. High‐tracking schools in Germany tend to provide their students with a deeper and more extended general education. In general, students attend classes with the same group of peers, with class sizes varying from 15 to 35 students. All participants were guaranteed the confidentiality of the data collection process and their voluntary involvement. Ethnicity data were not gathered due to the limited representation of ethnic minorities in the area (2.6%). Additionally, economic status information, such as family income, was not collected in compliance with German law, which prohibits participants from disclosing any personal information about another person, including parental income. Instructors who underwent training introduced the paper–pencil questionnaire format and explained the utilization of Likert scales. They were also present during the data collection sessions.

### Measures

This study employed established self‐report instruments validated for German adolescent students. The original materials were in German and have been translated in this paper to offer examples for an international readership. Example items, ranges and reliability for both measurement points of the scales for peers as positive and negative motivators, student–student relationship, motivational self‐regulation and grades are presented in Tables 1, 2, 3.

**TABLE 1:** Measures peer relationships and student–student relationship.

Measure	Introduction	Example Items	Range	Cronbach Alpha
Peers as positive motivators – 9 items Raufelder, Drury, et al. (2013)	Please tick how much you agree with the following statements?	“When my friends learn, it motivates me to learn more.” “If my friends want to be better at school, then I want to be better too.”	(1) do not agree at all to (4) completely agree	$T_{1}\alpha =.79$; $T_{2}\alpha =.81$
Peers as negative motivators – 6 items Raufelder, Drury, et al. (2013)	Please tick how much you agree with the following statements?	“My friends pay more attention to me when I put less effort into school” “If my friends find school boring, then I often don't feel like going to school either.”	(1) do not agree at all to (4) completely agree	$T_{1}\alpha =.69$; $T_{2}\alpha =.73$
Student–student relationship – 6 items PISA; Kunter et al. (2002)	This is about your personal perception of the class.	“Many students are envious if others have better grades” “There are some pupils in our class who receive little attention from the others.”	(1) strongly disagree to (4) strongly agree	$T_{1}\alpha =.70$; $T_{2}\alpha =.71$

Abbreviations: T1, time 1 (beginning of eighth grade); T2, time 2 (end of ninth grade).

**TABLE 2:** Measures motivational self‐regulation.

Measure	Introduction	Example Items	Range	Cronbach Alpha
Intrinsic motivation – 5 items Müller et al. (2007)	I work and study at school because…	“… I enjoy it.” “… I want to learn new things.”	(1) it is not true at all to (5) it is absolutely true	$T_{1}\alpha =.79$; $T_{2}\alpha =.82$
Identified regulation – 4 items Müller et al. (2007)	See Intrinsic Motivation	“… it gives me more options when choosing a career later on” “… I can put the things I learn here to good use later on.”	(1) it is not true at all to (5) it is absolutely true	$T_{1}\alpha =.90$; $T_{2}\alpha =.92$
Introjected regulation – 4 items Müller et al. (2007)	See Intrinsic Motivation	“… I want my teacher to think I am a good student” “… I would have a guilty conscience if I did very little”	(1) it is not true at all to (5) it is absolutely true	$T_{1}\alpha =.69$; $T_{2}\alpha =.71$
External regulation – 4 items Müller et al. (2007)	See Intrinsic Motivation	“… otherwise, I get bad grades” “… otherwise, I get pressure from home.”	(1) it is not true at all to (5) it is absolutely true	$T_{1}\alpha =.66$; $T_{2}\alpha =.61$

Abbreviations: T1, time 1 (beginning of eighth grade); T2, time 2 (end of ninth grade).

**TABLE 3:** Measures grades.

Measure	Introduction	Example Items	Range	Cronbach Alpha
Grades – 4 items	What was your grade at the end of the last school year in this subject?	“Mathematics” “German” “English” “Biology”	(1) very good to (6) insufficient	$T_{1}\alpha =.81$; $T_{2}\alpha =.74$

Abbreviations: T1 = time 1 (beginning of eighth grade), T2 = time 2 (end of ninth grade).

### Statistical analyses

A latent cross‐lagged panel analysis was employed to examine the hypotheses, enabling the investigation of both within‐time and over‐time associations in the dynamics between students' motivational self‐regulation and students' social relations with peers. This method is extensively utilized to assess the stability and relationships between variables both within and over time, providing a deeper understanding of how these variables mutually influence each other (Kearney, 2017). Due to the substantial number of items (a total of 82 items combined of the two measure points) adversely impacting the model fit (Ding et al., 1995; Wang & Wang, 2012), we opted to leverage the advantages of parcelling, as suggested by Little et al. (2002) as well as Nasser and Wisenbaker (2003). This approach offers advantages in terms of both psychometric properties and model estimation and fit characteristics. Parcels exhibit higher reliability compared to item‐level data, a greater ratio of common‐to‐unique factor variance, and a reduced likelihood of distributional violations. Additionally, parcels feature more precise and evenly spaced intervals, fewer parameter estimates, a lower indicator‐to‐sample size ratio, a diminished probability of correlated residuals and dual factor loadings and decreased sources of sampling error (Little et al., 2002, 2013).

Based on Nasser and Wisenbaker (2003), items of all variables in this study were randomly split into two parcels each. The two PPM parcels consist of five and four items, while SSR and PNM parcels each consist of three items. INT parcels are very split into parcels of three and two, while IDEN, INJE, EXT and Grade parcels each consist of two items. To evaluate the model fit, four primary fit indices were used: (a) the Chi‐Square Test of Model Fit (*χ* ^2^), (b) the Comparative Fit Index (CFI), (c) the Root Mean Square Error of Approximation (RMSEA) and (d) the Standardized Root Mean Square Residuals (SRMR), reported in Table 2. In accordance with the recommendations by Hu and Bentler (1999), we considered TLI/CFI values close to .95, RMSEA values nearing .06 and SRMR values equal to or below .08 as indicative of a satisfactory model fit. To examine whether the stepwise application of measurement invariance constraints results in a significant decrease in model fit, the *χ* ^2^ difference test was employed. The test was computed using the Satorra–Bentler scaling correction factor (Satorra & Bentler, 2001) to assess the stability of factor loadings, intercepts and error terms across different time points. Mplus 7.0 (Muthén & Muthén, 2012) and robust maximum likelihood estimation with robust standard errors (MLR) and chi‐square values to assess our hypotheses were employed. The “Type is Complex” approach in Mplus was utilized to accommodate the nested structure of the data, with 779 students distributed across 47 classes, reflecting the stable class structure in the German school system from the seventh to the tenth grades. At T1, missing values of students' reports were low (ranging from 1%–4%). Due to the 1.5 years gap between the two measurements, the drop‐out rate was relatively large (ranging from 23%–27%–highest item‐based missing values on the student–student relationship scale). Full‐information maximum likelihood (FIML) estimation was applied for handling missing data, given that the missing data were completely random (MCAR), as confirmed by Little's MCAR test for all parcels (*χ* ^2^ = 517.42, df = 489, *p* > .05).

### Results

#### Descriptive statistics and bivariate correlations

The intercorrelations, ranges, means and standard deviations between all variables are reported in Table 4. In Appendix 1, Table A1, the intercorrelations, ranges, means and standard deviations between all variables aggregated on the class level are reported. Intraclass correlations (ICCs) are reported in Appendix 1 (Table A2).

**TABLE 4:** Means, standard deviations and latent correlations for the constructs.

Measure	2	3	4	5	6	7	8	9	10	11	12	13	14	15	16	Range	Mean	*SD*	*N*
1. PPM T1	.39**	.09*	.46	.11**	.48	.19**	.10*	.12**	.15**	.29**	.22**	.11**	.12**	.01	−.05	14	2.60	.50	779
2. PPM T2		.08	.12**	.07	.09*	.13**	.17**	.11*	.12**	.19**	.34**	.11*	.14**	.04	−.03	1–4	2.52	.49	600
3. PNM T1			.42**	.15**	.06	−.17**	−.14**	−.11**	−.10*	.03	−.05	.05	.01	.15**	.16**	1–4	1.49	.39	779
4. PNM T2				.09*	.16**	−.16**	−.26**	−.05	−.11**	.03	.01	.12**	.10*	.14**	.17**	1–4	1.55	.43	600
5. SSR T1					.47**	.02	−.03	.01	.05	.23**	.15**	.15**	.11**	.09*	.07	1–4	2.31	.52	761
6. SSR T2						.06	−.05	.07	.06	.18**	.22**	.11*	.21**	−.01	−.00	1–4	2.37	.55	565
7. INT T1							.41**	.36**	.09*	.33**	.18**	.06	−.01	−.10**	−.17**	1–5	4.05	.73	752
8. INT T2								.15**	.30**	.07	.22**	−.05	−.07	−.12**	−.16**	1–5	4.11	.72	580
9. IDEN T1									.35**	.31**	.17**	.34**	.06	−.09**	−.15**	1–5	3.52	1.09	752
10. IDEN T2										.05	.19**	.16**	.26**	−.10*	−.12**	1–5	3.46	1.19	580
11. INJE T1											.43**	.52**	.23**	.00	−.04	1–5	2.89	.86	752
12. INJE T2												.27**	.48**	−.01	−.00	1–5	2.95	.91	580
13. EXT T1													.37**	.07	.13**	1–5	2.45	.82	752
14. EXT T2														.00	.06	1–5	2.37	.77	580
15. Grades T1															.65**	1–5	2.23	.64	766
16. Grades T2																1–4	2.45	.61	588

Abbreviations: EXT, external regulation; IDEN, identified regulation; INJE, introjected regulation; INT, intrinsic motivation; PNM, peers as negative motivators; PPM, peers as positive motivators; SSR, student–student relationship; T1, time 1 (eighth grade); T2, time 2 (ninth grade).

**p < .01, *p < .0.

#### Measurement invariance test

Before conducting the latent cross‐lagged panel design, we tested the measurement invariance of all latent variables built by the parcels over time using the following steps: (1) specifying an unconditional model (configural invariance) without equality constraints, (2) specifying the factor loadings as invariant over time (weak factorial invariance) and (3) setting the loadings and item intercepts as invariant over time (strong factorial invariance) (Table 5). The results indicate strict measurement invariance over time, suggesting that all the constructs remained stable, thereby allowing the application of the cross‐lagged panel design.

**TABLE 5:** Summary of results for tests of longitudinal measurement invariance.

Model	df	*χ* ^2^	*p*	CFI	TLI	RMSEA	90% CI	SRMR	ΔCFI	ΔTLI	ΔRMSEA	ΔSRMR
1 configural MI	252	475.23	<.001	.963	.944	.034	[.029–.038]	.037	–	–	–	–
2 metric MI	259	478.96	<.001	.963	.946	.033	[.028–.038]	.037	0	.002	.001	0
3 scalar MI	266	520.10	<.001	.957	.940	.035	[.031–.039]	.038	.006	.006	.002	.001

*Note*: MI, measurement invariance; the final columns indicate model fit comparisons between the two subsequent models. (Chen, 2007): ΔCFI = decrease of ≤.010, ΔSRMR = decrease of ≤.010, ΔRMSEA = increase of ≤.015.

#### Latent cross‐lagged panel analysis

**FIGURE 1:**
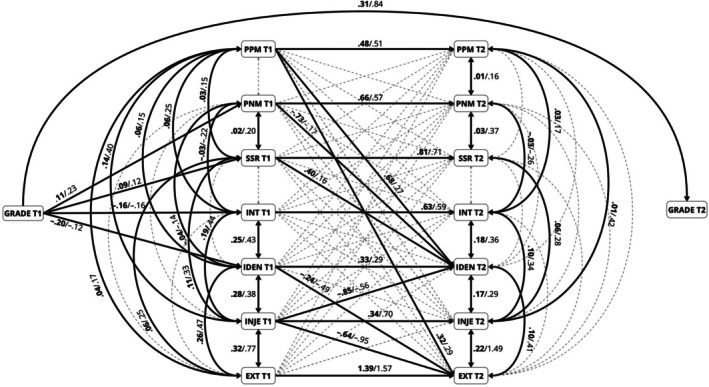
Cross‐lagged panel design between PPM, PNM, SSR and the four motivational regulations. Significant effects are shown as unstandardized coefficients (*B*) in boldface and standardized coefficients (*β*) in italics. Bold pathways are significant at *p* < .05; dotted pathways are not significant. EXT, extrinsic motivation; IDEN, identified regulation; INJE, introjected regulation; INT, intrinsic motivation; PNM, peers as negative motivators; PPM, peers as positive motivators; SSR, student–student relationship; T1, time 1 (beginning of eighth grade); T2, time 2 (end of ninth grade).

The final cross‐lagged model (see Figure 1) demonstrated a good fit: *χ* ^2^ (358) = 894.80, *p* < .001; CFI = .93, RMSEA = .04 (.04–.05); SRMR = .04. Given that the *χ* ^2^ value is sensitive to sample size and “nearly always rejects the model when large samples are used” (Bentler & Bonett, 1980; Hooper et al., 2008; Jöreskog & Sörbom, 1993, p. 54), its significance in this study can be disregarded as it is not a reasonable measure of fit for models with 400 or more cases (Bentler & Bonett, 1980; Kline, 2011; Schumacker & Lomax, 2004). The standardized factor loadings of this model are presented in Appendix 1 (Table A3).

#### Within‐time associations

Covariances between all constructs within‐time at T1 (beginning of eighth grade) and T2 (end of ninth grade) are shown in Tables 6 and 7.

**TABLE 6:** Results of the cross‐lagged panel model. Within‐time associations time 1 (beginning of eighth grade).

Measure	2	3	4	5	6	7	8
1. PPM T1	.02/.13	.03/.15*	.06/.25*	.06/.15*	.14/.40*	.04/.17*	.01/.01
2. PNM T1		.02/.20*	−.03/−.22*	−.04/−.14*	.00/.03	.01/.07	.11/.23*
3. SSR T1			.01/.05	.02/.05	.11/.33*	.06/.25*	.09/.12*
4. INT T1				.25/.43*	.19/.44*	.03/.11	−.16/−.16*
5. IDEN T1					.28/.38*	.26/.47*	−.20/−.12*
6. INJE T1						.32/.77*	−.01/−.01
7. EXT T1							.09/.09
8. Grades T1							

*Note*: Significant effects *(*p* < .05) are shown as unstandardized coefficients (B) in boldface and standardized coefficients (*β*) in italics.

Abbreviations: EXT, external regulation; IDEN, identified regulation; INJE, introjected regulation; INT, intrinsic motivation; PNM, peers as negative motivators; PPM, peers as positive motivators; SSR, student–student relationship; T1, time 1 (eighth grade).

**TABLE 7:** Results of the cross‐lagged panel model. Within‐time associations time 2 (end of ninth grade).

Measure	2	3	4	5	6	7	8
1. PPM T2	.01/.16*	.02/.15	.03/.17*	.01/.04	.01/.42*	.01/.09	.00/.00
2. PNM T2		.03/.37*	−.03/−.26*	−.03/−.14	.01/.07	.01/.18	.05/.10
3. SSR T2			−.02/−.10	−.01/−.02	.06/.28*	.08/.92	−.02/−.03
4. INT T2				.18/.36*	.10/.34*	−.04/−.34	−.07/−.07
5. IDEN T2					.17/.29*	.10/.41*	−.19/−.10
6. INJE T2						.22/1.49*	.06/.05
7. EXT T2							−.04/−.05
8. Grades T2							

*Note*: Significant effects *(*p* < .05) are shown as unstandardized coefficients (B) in boldface and standardized coefficients (*β*) in italics.

Abbreviations: EXT, external regulation; IDEN, identified regulation; INJE, introjected regulation; INT, intrinsic motivation; PNM, peers as negative motivators; PPM, peers as positive motivators; SSR, student–student relationship; T2, time 2 (ninth grade).

#### Over time associations

All significant autoregressive paths from one construct to itself and the cross‐lagged paths over time are presented in Table 8.

**TABLE 8:** Results of the cross‐lagged panel model. Over‐time associations.

Measure	PPM T2	PNM T2	SSR T2	INT T2	IDEN T2	INJE T2	EXT T2
1. PPM T1	.48/.51*	.02/.02	−.08/−.07	.13/.10	.68/.27*	.12/.07	.32/.29*
2. PNM T1	.07/.51	.65/.57*	−.10/−.05	−.19/−.08	−.73/−.17*	−.33/−.12	−.19/−.10
3. SSR T1	−.01/−.01	−.01/−.02	.81/.71*	.00/.00	.40/.16*	.03/.02	.07/.06
4. INT T1	.08/.11	−.01/−.01	−.08/−.09	.63/.59*	.10/.05	−.11/−.09	.31/.36
5. IDEN T1	−.01/−.03	.00/.00	.08/.16	−.04/−.07	.33/.29*	.02/.03	−.24/−.49*
6. INJE T1	−.07/−.13	−.07/−.16	.15/.22	−.22/−.26	−.85/−.56*	.34/.70*	−.64/−.95*
7. EXT T1	.14/.19	.12/.21	−.24/−.27	.15/.14	.85/.42	−.19/−.14	1.39/1.57*
	Grades T2						
8. Grades T1	.31/.84*						

*Note*: Significant effects *(*p* < .05) are shown as unstandardized coefficients (B) in boldface and standardized coefficients (*β*) in italics.

Abbreviations: EXT, external regulation; IDEN, identified regulation; INJE, introjected regulation; INT, intrinsic motivation; PNM, peers as negative motivators; PPM, peers as positive motivators; SSR, student–student relationship; T1, time 1 (eighth grade); T2, time 2 (ninth grade).

## DISCUSSION

This study aimed to test the potential reciprocal relationship between mid‐adolescent students' motivation and their perceived peer relationships in class by considering (a) different facets of peer relationships, (b) the peculiarities of peer relationships in high‐track schools and (c) the quality of motivation in a differentiated way. Using cross‐lagged panel analysis, this study investigated potential reciprocal associations between peer social relationships and the quality of motivation in a school context. It utilized two waves of data from adolescent students, spanning the beginning of eighth grade through the end of ninth grade.

H1a was partly confirmed. A high‐quality student–student relationship was positively related to students' identified regulation 1.5 years later. This aligns with SDT (Ryan & Deci, 2000), which posits that feelings of relatedness within social contexts support the internalization of values, fostering identified regulation. However, the absence of a significant association with introjected and external regulation suggests that close peer relationships may not inherently exert the types of normative or external pressures needed to trigger these motivational states. The lack of a link between student–student relationships and intrinsic motivation aligns with the hypothesis and the existing literature. Intrinsic motivation, being entirely self‐driven, may depend more on individual interests and internal satisfaction than on interpersonal dynamics (Ryan & Deci, 2000). This distinction underscores that even positive peer relationships, while important, may primarily support more controlled forms of motivation rather than fully autonomous motivation.

H1b was also partly confirmed. The perception of peers as positive motivators was positively associated with introjected and identified regulation, both concurrently and over time. This finding suggests that positive peer influence reinforces internalized goals and a sense of competence (Ryan, 2001). However, the absence of a relationship with intrinsic motivation highlights a limitation of external motivational sources: even when peers are supportive, intrinsic interest arises independently of social validation.

Surprisingly, H1c was only partly supported. While it predicted that peers as positive motivators would not relate to external regulation, the results showed a significant positive association. This unexpected finding could indicate that even supportive peers may occasionally introduce pressure to conform to group norms, leading to compliance‐driven motivation. However, this effect may differ depending on the nature of peer influence, such as whether it emphasizes encouragement or competition. By contrast, as hypothesized, student–student relationships did not significantly relate to external motivation, likely reflecting the more intrinsic nature of such relationships compared to the potentially goal‐oriented influence of motivator peers.

H2a was partly confirmed. Peers perceived as negative motivators were negatively associated with identified regulation, both concurrently and over time. This result supports the premise that negative peer influences undermine feelings of relatedness and competence, thereby hindering internalized forms of motivation (Ryan & Deci, 2000). However, the absence of significant associations with introjected regulation or intrinsic motivation suggests that negative peer influences may primarily disrupt motivation related to self‐determined goals rather than affect more externally driven or self‐sustaining forms of motivation.

Contrary to H2b, no significant relationship was found between negative motivators and external regulation. A potential explanation is that external regulation may require explicit pressures or tangible consequences (e.g., grades or disciplinary actions) rather than implicit negative peer dynamics. Alternatively, it is possible that the negative motivators in this study were insufficiently strong to provoke the compliance or avoidance behaviours typically linked to external regulation (Veenstra & Laninga‐Wijnen, 2022). Further research could explore the intensity and context of negative peer influences to clarify these relationships.

In sum, the results confirm the assumption that peer influences on motivation act primarily in the middle of the spectrum of externally regulated gradations (Beiswenger & Grolnick, 2009; except for the path from peers as positive motivators predicting external regulation, as discussed below), since intrinsic motivation is by nature independent of external influences, and pure extrinsically motivated behaviour is more affected by avoidance of punishment or the search for praise, which may be influenced more by teachers or parents.

The fact that all three peer variables significantly impact identified regulation over time might indicate that the greatest influence can be observed here: if the individual perceives peers as positive motivators or experiences positive student–student relationships in class, then these peers might also function as role models or examples, demonstrating to the individual that motivation is a matter of personal significance (identified regulation). Positive peer relationships can create environments in which peers discuss their goals, share the relevance of academic tasks to their personal aspirations and support each other's engagement, thereby promoting identified regulation. They might also fulfil the basic need of relatedness and, as such, contribute to students' self‐determination (Katz & Assor, 2007; Niemiec & Ryan, 2009).

Another explanation could be Bandura & Walters (1977) social learning theory, which emphasizes that individuals learn through observing others' attitudes, behaviours and the consequences of those behaviours. Peers who demonstrate strong internalized motivation by valuing learning and persisting through challenges can serve as models. Their actions show the benefits of embracing long‐term goals and personal values, influencing others to internalize similar attitudes towards their own schoolwork.

Conversely, if individuals experience peers as negative motivators who suggest that motivation is irrelevant to oneself, this will likely prevent higher autonomous forms of motivation (identified regulation). In this case, the need for social relatedness is satisfied; however, the meaningfulness of school motivation for one's own self is questioned, which might be associated with a sense of alienation from school (Morinaj et al., 2021). This finding also shows that it is not sufficient to merely enquire about the fulfilment of the need for relatedness with peers: it is also necessary to consider the standards, norms and attitudes of the peer group in the sense of a secondary socialization instance (Nickerson et al., 2022; Ryan, 2001). Future studies, particularly qualitative studies, could provide deeper insights into how different forms of peer support might differently affect the specific forms of external regulation.

Interestingly, only peers as positive motivators were positively associated with external regulation over time. This could signal a kind of “dependency” (Jagenow et al., 2015; Raufelder, Jagenow, et al., 2013) in which students link their motivation externally to their peers and are then no longer able to motivate themselves without peers. Such a dependency is, of course, unfavourable because peers as motivators should function more as external sources that favour the quality of motivation towards more autonomous forms of motivation. In other words, teachers should ensure that peer support is provided in a way that encourages motivation so that students are still supported in their autonomy – in the sense of helping them to help themselves (as indicated in the model by the significant path between peers as positive motivators and identified regulation). Another explanation for this finding could be that peers adopt this controlled form of motivation from everyday school life in secondary schools, which are known to involve more extrinsic controls and rigid constraints (Ratelle et al., 2007). Peers would then teach an individual with peer dependency to do only what is necessary to avoid punishment and receive rewards because this is the motivational concept they have been taught and adopted at school (Ratelle et al., 2007).

In contrast to our hypotheses, the interplay of peer relationships and motivational regulation was not reciprocal, which could indicate a potential causal order, such as peer support fostering motivation but not the other way around. Future longitudinal studies with additional measurement points are necessary to validate this assumption. This result would contradict the findings that high motivation can benefit more positive social behaviours (e.g., Eccles & Wigfield, 2002; Fredricks et al., 2004; Hattie et al., 2020; Kilday & Ryan, 2022). However, our variables do not cover the full range of positive social behaviours but are very specific to motivational support and the quality of social relationships. Here, too, follow‐up studies that consider other social behaviours (e.g., prosocial behaviour) and several measurement points within shorter time intervals (e.g., within a school year) could provide more information.

H3a and H3b were partly confirmed: grades were positively associated concurrently with peers as negative motivators and with student–student relationships. For instance, the worse the grades, the more students tend to perceive their peers as negative motivators (H3b) and the more they perceive better student–student relationships, and vice versa (H3a). Joining peer groups that have negative attitudes towards school motivation when one does not perform so well can be a kind of protective mechanism. One looks for like‐minded people so that poor grades become “normal”, in comparison with others in the peer group, and not a sign of weakness or lack of competence and intelligence, which one could otherwise unfavourably attribute (Weiner, 1986) to oneself or, in the worst case, integrate into oneself, leading to a negative self‐concept in school. In this respect, peers act as anchors who can at least fulfil the need for social integration. However, the reverse can also be true, with the peer group promoting poor grades by acting as negative motivators. Previous studies underline this outcome: peers with negative affect towards school tend to negatively affect students' school satisfaction (Ryan, 2000).

At first glance, it may seem surprising that lower grades are associated with better student–student relationships. At second glance, this may be explained by the highly competitive nature of high‐track schools: students with better grades may perceive their peers more as competitors and therefore collaborate less with them. A national survey of German youth revealed that most place growing significance on “standing out from the crowd” by emphasizing their personal uniqueness and individuality (Fritzsche, 2000). Consequently, the connections between individuals are weak, with each person being expected to take care of themselves and their immediate family, bearing responsibility for their academic achievements and career (Hesse, 2004).

Grades were further negatively associated with intrinsic and identified regulation; for instance, the worse the grades, the less students report intrinsic and identified regulation, and vice versa. This is in line with previous studies, which have shown correlations between motivational regulation and grades (Shao et al., 2024; Wentzel, 1998). However, it is also obvious that if students find learning content personally meaningful or are intrinsically absorbed in their schoolwork, they will generally also achieve better results in performance tests.

Our findings support the assumption that peer influence plays a role in shaping students' academic motivation, which aligns with Social Learning Theory (Bandura & Walters, 1977). However, other theoretical perspectives, such as Identity Theory (Brechwald & Prinstein, 2011) and Peer Pressure Theory (Veenstra & Laninga‐Wijnen, 2022), also provide relevant explanations for how peer influence processes unfold. Identity Theory suggests that motivation may be shaped by students identifying with academically engaged or disengaged peers, while Peer Pressure Theory highlights the role of explicit social reinforcement or exclusion fears in shaping behaviour. While our study does not explicitly differentiate between these mechanisms, our findings align most closely with Social Learning Theory, as they indicate that students' motivation is shaped by the behaviours and attitudes of their peers through observation and reinforcement. Future studies could further investigate the relative contributions of these different peer influence mechanisms.

### Strength, limitations and future directions

**FIGURE A1:**
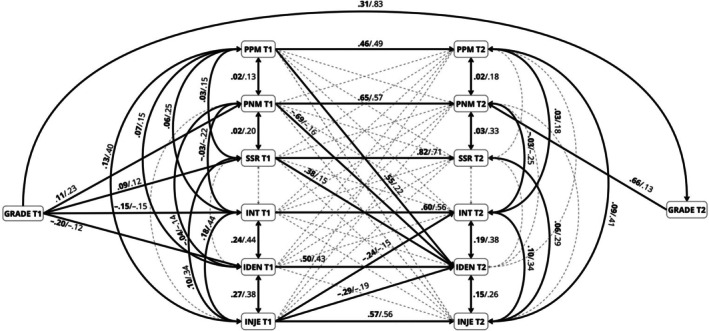
Cross‐lagged panel design between PPM, PNM, SSR and the three motivational regulations (INT, IDEN, INJE). Significant effects are shown as unstandardized coefficients (B) in boldface and standardized coefficients (*β*) in italics. Bold pathways are significant at *p* < .05; dotted pathways are not significant. Model‐Fit: *χ* ^2^ (271) = 848.99, *p* < .001; CFI = .94, RMSEA = .04 (.04–.05); SRMR = .04. IDEN, identified regulation; INJE, introjected regulation; INT, intrinsic motivation; PNM, peers as negative motivators; PPM, peers as positive motivators; SSR, student–student relationship; T1, time 1 (beginning of eighth grade); T2, time 2 (end of ninth grade).

This study has some methodological limitations. Using questionnaires relied on self‐reported data, capturing students' perceptions of motivational regulation, social relationships and grades. While self‐reported data are subjective, they were appropriate given the study's focus on internal states and adolescents' ability to articulate them. Although self‐reported grades may lack objectivity, they correlate well with actual grades and provide insights into self‐concept, motivation and self‐assessment (Shaw & Mattern, 2009). Future studies could include students from low‐track schools to identify differences in motivational development systematically. Additionally, SDT may not fully capture the complexity of students' motivational processes, due to an overall low level of intrinsic motivation. Future research should consider complementing it with alternative theoretical perspectives or mixed‐methods approaches to gain a more comprehensive understanding of adolescent motivation. Furthermore, the external regulation scale showed a low Cronbach's alpha (Koop & Lois, 2012), reflecting limited reliability. To address this, the CLPM was rerun without this variable (Figure A1). Another limitation is the study's two measurement points within 1.5 years; more frequent measures could better capture peer relationship dynamics and motivational regulation development. The results also could have been influenced by changes in class composition. Such changes may have impacted the stability of these relationships and the interpretation of our results. Although class composition dynamics were not directly accounted for in the study design, future research could explore how these changes influence peer relationships by considering which classes experienced substantial shifts and how these changes occurred. Future research could also explore latent growth models or the influence of perceived peer relationships on motivational changes. Integrated regulation was not addressed due to methodological challenges (Ryan & Deci, 2000), but future studies could explore this dimension further. Additionally, social network analyses (e.g., BJEP‐2024‐0151; BJEP‐2024‐0157; BJEP‐2024‐0213; BJEP‐2024‐0215; BJEP‐2024‐0384) could provide a more detailed understanding of peer influence at the dyadic level, allowing researchers to assess whether certain individuals are more susceptible to peer influence (e.g., unpopular adolescents) or whether some peers exert greater influence than others (e.g., popular or well‐liked adolescents). Finally, another limitation is the parcelling process in this study, as it may obscure item‐level variability and the dimensionality of the construct. While parcelling simplifies the model, it may overlook the nuances of peer interactions. Future studies could consider item analysis to address this limitation.

Despite limitations, the study has significant strengths. It involved a large sample and repeated data collection, enabling generalizability in Brandenburg, Germany. It also considered multiple aspects of peer relationships and motivation, using cross‐lagged panel analysis to examine patterns, directional effects and construct stability (Burkholder & Harlow, 2003) over time.

In sum, fostering positive peer relationships in school is essential for enhancing students' motivation, particularly through identified regulation, which involves students recognizing the value and importance of their learning goals and aligning them with their personal values. This type of motivation is more likely to lead to sustained engagement and deeper academic involvement.

To support this process, teachers should actively cultivate a classroom environment that fosters supportive peer interactions while minimizing dependency on external regulation. If peer relationships primarily reinforce external motivators such as grades or social approval, students may become more extrinsically driven, which could undermine long‐term engagement. When peers act as positive motivators, they should encourage autonomy and personal growth rather than reinforcing external dependencies.

Furthermore, positive social relationships and academic achievement are often interconnected, particularly in high‐achieving environments where performance pressures are common. While peer motivation can inspire academic effort, it may also contribute to stress and anxiety if success becomes a social expectation rather than an intrinsic goal. Teachers should therefore strive to balance the promotion of positive peer interactions with strategies that reduce excessive performance pressures, thereby protecting students' intrinsic motivation and well‐being.

## AUTHOR CONTRIBUTIONS

**Fabian Schimmelpfennig:** Formal analysis; writing – original draft; writing – review and editing.

## CONFLICT OF INTEREST STATEMENT

The authors declare no conflicts of interest.

## Data Availability

Both the data and study material are not available in any open source but can be accessed by contacting the author.

## APPENDIX 1

**TABLE A1:** Means, standard deviations and latent correlations for the aggregated constructs.

Measure	2	3	4	5	6	7	8	9	10	11	12	13	14	15	16	Range	Mean	*SD*
1. PPM T1	.55**	−.07*	−.42	−.09**	−.07*	.40**	.36**	.15**	.16**	.16**	.35**	−.16**	−.15**	−.25**	−.20**	1.7–2.9	2.55	.21
2. PPM 21		.08**	.11**	−.01	.08**	.12**	.39**	.13**	.28**	.22**	.32**	.17**	.10**	−.31**	−.22**	1.2–3.0	2.46	.22
3. PNM T1			.56**	.32**	.13**	−.42**	−.44**	−.16**	−.06	.15**	−.04	.34**	.33**	.15**	.16**	1.0–1.8	1.54	.17
4. PNM T2				.34**	.14**	−.14**	−.39**	.04	−.16**	.36**	.09**	.47**	.36*	.29**	.34**	1.1–1.8	1.57	.26
5. SSR T1					.32**	−.08**	−.36**	.07*	−.09**	.30**	−.05	.34**	.23**	.40**	.25**	2.0–2.6	2.35	.18
6. SSR T2						−.15**	−.09**	−.08**	.11**	.09**	.18**	.18**	.46**	.04	.04	1.9–2.9	2.40	.23
7. INT T1							.52**	.62**	.27*	.20**	.23**	−.33**	−.31**	−.20**	−.36**	3.5–4.8	4.00	.28
8. INT T2								.27**	.49**	−.17**	.31**	−.36**	−.26**	−.53**	−.48**	3.5–4.7	4.01	.32
9. IDEN T1									.36**	.34**	.22**	.14**	.02	−.10**	−.21**	2.8–4.6	3.50	.31
10. IDEN T2										−.04	.33**	.06	.27**	−.32*	−.29**	2.6–4.6	3.44	.44
11. INJE T1											.34**	.51**	.19**.00	.17**	.15**	2.0–3.7	2.89	.24
12. INJE T2												.18**	.53**	−.05	−.06*	1.8–3.9	2.93	.31
13. EXT T1													.52**	.20**	.38**	1.9–3.0	2.48	.26
14. EXT T2														.23**	.27**	1.8–3.0	2.43	.28
15. Grades T1															.71**	1.4–2.9	2.42	.46
16. Grades T2																1.7–2.9	2.58	.35

Abbreviations: EXT, external regulation; IDEN, identified regulation; INJE, introjected regulation; INT, intrinsic motivation; PNM, peers as negative motivators; PPM, peers as positive motivators; SSR, student–student relationship; T1, time 1 (eighth grade); T2, time 2 (ninth grade).

***p* < .01, **p* < .05.

**TABLE A2:** Intraclass correlations for the constructs.

Measure	*ICC* (*T1/T2*)
PPM	.068/.027
PNM	.059/.038
SSR	.057/.069
INT	.097/.065
IDEN	.038/.035
INJE	.019/.017
EXT	.053/.022

Abbreviations: EXT, external regulation; IDEN, identified regulation; INJE, introjected regulation; INT, intrinsic motivation; PNM, peers as negative motivators; PPM, peers as positive motivators; SSR, student–student relationship; T1, time 1 (eighth grade); T2, time 2 (ninth grade).

**TABLE A3:** Range of standardized factor loadings $\left(\text{λmin}.-\max.\right)$ for latent factors.

Latent factor	*T1*	*T2*
Peers as positive motivators	.77–.80	.75–.82
Peers as negative motivators	.62–.74	.66–.84
Student–student relationship	.67–.76	.61–.81
Intrinsic motivation	.81–.81	.79–.89
Identified regulation	.85–.91	.91–.92
Introjected regulation	.75–.79	.75–.78
Extrinsic motivation	.64–.68	.56–.63
